# Donor site morbidity following radial forearm free flap reconstruction with split thickness skin grafts using negative pressure wound therapy

**DOI:** 10.1186/s40463-019-0344-9

**Published:** 2019-05-21

**Authors:** Jessica M. Clark, Shannon Rychlik, Jeffrey Harris, Hadi Seikaly, Vincent L. Biron, Daniel A. O’Connell

**Affiliations:** 1 Department of Surgery, Division of Otolaryngology-Head and Neck Surgery, University of Alberta, 8440-112 St, 1E4 Walter Mackenzie Centre, Edmonton, Alberta T6G 2B7 Canada; 2 Alberta Head and Neck Centre for Oncology and Reconstruction, Edmonton, Alberta Canada

**Keywords:** Radial forearm free flap, Split thickness skin graft, Negative pressure wound therapy, Wrist, Hand, Patient-reported outcomes

## Abstract

**Background:**

Donor site complications secondary to radial forearm free flap (RFFF) reconstruction can limit recovery. Optimizing hand and wrist function in the post-operative period may allow more efficient self-care and return to activities of daily living. Negative pressure wound dressings (NPD) may increase blood flow and perfusion as compared to static pressure dressings (SPD) designed to minimize shear forces during the healing period. This study aims to compare subjective and objective hand and wrist functional outcomes following RFFF reconstruction with split thickness skin grafts (STSG) in patients treated with NPD and SPD.

**Methods:**

Adult patients undergoing RFFF with STSG were identified preoperatively and randomized to receive NPD or SPD following their RFFF reconstruction. NPD involved a single-use, portable device capable of applying 80 mmHg of negative pressure to the forearm donor site. SPD involved a volar splint. Dressings were left in place for seven days with subjective and objective function assessed at seven days, one month and three months postoperatively. The primary outcome was self-reported hand function as measured with the function subscale of the Michigan Hand Questionnaire (MHQ). Secondary outcomes included hand and wrist strength, range of motion, sensation, scar aesthetics, and skin graft complications.

**Results:**

Twenty-four patients undergoing RFFF were randomized to NPD or SPD. Patients treated with NPD had improved MHQ self-reported functional scores as compared to those treated with SPD at seven days postoperatively (*P* = 0.016). Flexion at seven days was improved in NPD group (*P* = 0.031); however, all other strength and range of motion outcomes were similar between groups. There were no differences in rates of graft complications, scar aesthetics, or sensation.

**Conclusions:**

In the immediate post-operative period, NPD was associated with improved patient-reported hand and wrist function. Wound care to optimize hand and wrist function could allow for improved patient outcomes in the immediate postoperative period.

## Background

Radial forearm free flaps are the most frequently used free tissue transfer for reconstruction of soft tissue defects in the head and neck following oncologic resections. The overall goal of reconstruction should be to maximize the function and cosmetic outcomes of the defect closure, while also minimizing the morbidity at the donor site. Forearm donor sites can rarely be closed primarily and typically require a split thickness skin graft for closure. Unfortunately, there are high rates of poor or delayed wound healing with resultant tendon exposure and skin graft failure [1–4] at the forearm donor sites.

Negative pressure wound therapy is effective for management of chronic and infected trauma and surgical wounds. Molecular evidence suggests that NPD improves blood flow and promotes the production of granulation tissue within the wound bed [5]. Several retrospective studies have found NPD to result in fewer graft-related complications as compared to traditional bolster dressings [6, 7] for head and neck cancer patients. The justification for the additional costs and supplies for the negative pressure system remains a barrier for universal implementation of these systems and there is not enough evidence to identify which patients would benefit from the additional healing potential.

Patient and nursing satisfaction with portable, single-use NPD is high [8]. Patients are pleased with the simple, portable nature of the device and report very normal function relating to activities of daily living while the dressing is in situ [9]. Health care professionals report the devices are easy to monitor for any vascular insufficiency and allows patients to functionally utilize their hands immediately postoperatively. The downside to all wound therapy remains the additional material costs. However, recently it was questioned if the potential improved patient outcomes and reduced costs associated with nursing care and shorter hospital stay could portend their additional up front costs [10]. A recent Cochrane review did identify the need for suitably powered, high quality trials to evaluate the use of NPD for use on clean, closed surgical wounds [11]. To date, there has been no randomized control trial to assess patient satisfaction as a primary outcome for traditional bolster dressings and NPD in patients undergoing free tissue transfer for head and neck oncologic reconstruction.

The primary objective of this trial was to determine if patients have improved subjective hand and wrist function with NPD as compared to a traditional bolster dressing. Secondary outcomes examined function, strength, sensation and range of motion of wrist before and after surgery, scar aesthetics and assessed the graft-related complications of both dressings.

## Methods

Institutional approval for the study was obtained from the University of Alberta health research ethics board. Consecutive, consenting patients undergoing RFFF for head and neck oncologic reconstruction over the age of 18 years were prospectively enrolled and randomized to receive a traditional bolster SPD or a portable NPD (PICO™ Smith & Nephew, Hull, UK). Coin flip randomization was performed to enroll patients until the targeted sample size was reached. Patients previously treated with free tissue transfer for head and neck reconstruction and those with a preoperative history of motor or sensory deficits distal to the proposed free flap were excluded from the study. The primary outcome was the “Overall Hand Function” subscale of the MHQ, which has a minimal clinically important difference of 12 [12]. Information from previous work at our centre examining functional and cosmetic outcomes in patients with RFFF [13] was utilized to performed a power calculation. With a two-sided test and a significance level of α = 0.05, 6 patients per arm would be required for a statistically significant difference with a power of 80% (1-β = 0.8). We elected to recruit 12 patients per arm to allow for dropouts and to improve the statistical power of our secondary outcomes.

### Questionnaires

#### Subjective hand function

The Michigan Hand Questionnaire is a simple and reliable instrument, which differentiates handedness and measures patient-reported outcomes for the hand and wrist. It has been shown to be reliable and effective at differentiating function over time [14]. There are six distinct scales – overall hand function, activities of daily living, pain, work performance, aesthetics and patient satisfaction.

#### Scar aesthetics

The Patient and Observer Scar Assessment Scale (POSAS) is a reliable and consistent tool used to assess scar quality [15]. It has been used in head and neck cancer patients [16, 17]and incorporates a patients’ opinion as well as the expert observer.

Preoperatively patients completed the MHQ, POSAS, and completed baseline range of motion (ROM) and strength testing using a goniometer and dynamometer.

**Fig. 1:**
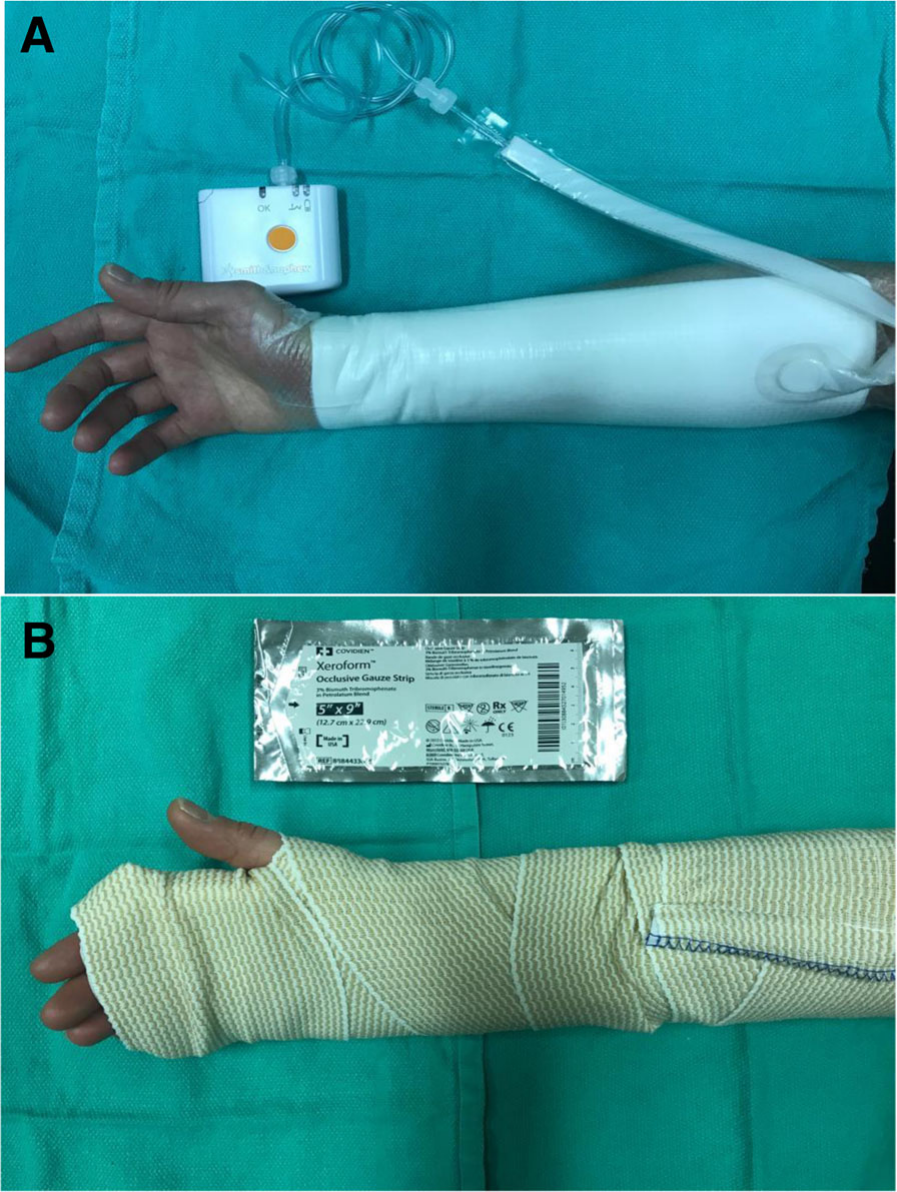
Application of negative pressure dressing (**a**) and static pressure dressing (**b**)

At the time of surgery, all forearm donor sites were covered using a STSG (0.016 in. thickness) from the thigh. STSGs were pie crusted, avoiding placing of defects overlying tendons; and sutured to surrounding skin using 4.0 monocryl. Patients in the NPD group had a single layer of petroleum-based meshed gauze placed over the STSG followed by the PICO system (Fig. 1a). PICO is a single use, portable device that provides 80 mmHg of negative pressure over the entire graft site. No immobilization splint was used in the NPD group. Patients in the SPD group had a thick layer of petroleum-based meshed gauze placed over the STSG, followed by dry gauze, cling and the wrist immobilized with a volar splint (Fig. 1b). Both dressings were applied under sterile technique in the operating room and removed seven days postoperatively.

At seven days postoperatively, an occupational therapist assisted patients to complete the function and activities of daily living subscale of the MHQ prior to removal of the dressings. The percentage of graft take was measured using a 1cmx1cm transparent grid. Any tendon exposure was also identified. Patients and observers completed the POSAS and the occupational therapist completed objective ROM and strength testing. Occupational therapists were not blinded to the treatment arm.

Subsequent follow up visits were done as outpatients and evaluators were blinded to the treatment arm. At one month and three months postoperatively, patients completely the POSAS, MHQ and underwent ROM and strength testing. Any graft complications were recorded at follow up visits.

Statistical analysis was completed using SPSS version 25.0 (SPSS Inc., Chicago, IL, USA) using chi squared and student t-testing for comparisons of means. Testing of normal distribution was confirmed and statistical significance was defined as *p* < 0.05. The researcher (JMC) who completed the analysis was blinded to the treatment arm throughout the study and analysis.

## Results

Twenty-four patients were included, with 12 patients randomized to SPD and 12 randomized to NPD. Patient demographics are described in Table 1. A higher proportion of males were included in the SPD group; however, there were no other significant differences between the groups. No patients were lost to follow up, with all patients completing one week, one month and three month follow up evaluations.

**Table 1:** Patient demographics

	SPD (SD)	NPD (SD)	Significance (*p* value)
Age (yr)	59.3 (11.8)	59.7 (12.6)	0.93
Sex (% male)	80%	33%	0.01
Comorbidities (n)	None: 5	None: 5	0.141
	1–2: 5	1–2: 3	
	3 or more: 2	3 or more: 4	
Smokers (%)	50%	45%	0.83
Stage	I: 2	I: 3	0.5
	II: 3	II: 6	
	III: 3	III: 1	
	IV: 3	IV: 2	
Graft size (cm^2^)	87.9 (44.3)	70.7 (24.9)	0.275

*SPD* static pressure dressing, *NPD* negative pressure dressing, *SD* standard deviation

All patients had normal self-reported hand function prior to surgery (MHQ = 100). Patients treated with NPD had significantly better self-reported function for their operated hand at seven days postoperatively (*P* = 0.016) (Table 2). This difference exceeded the minimal clinically important difference of the MHQ function subscale by more than two-fold. There were no differences that were retained in this subscale at one or three month postoperatively and no differences were observed for other subscales of the MHQ at all follow up visits.

**Table 2:** Overall hand function on the Michigan Hand Questionnaire comparing negative and static pressure dressings. MHQ scored from 0 to 100 with 100 representing normal hand and wrist function

	SPD (SD)	NPD (SD)	Significance (p value)
7 days	43.3 (28.5)	70.8 (22.7)	0.02
1 month	64.5 (16.6)	69.2 (17.7)	0.52
3 months	70.5 (15.2)	83.8 (17.3)	0.06

*SPD* static pressure dressing, *NPD* negative pressure dressing, *SD* standard deviation

Patients treated with NPD had improved ROM, as demonstrated with improved flexion at 7 days postoperatively (*P* = 0.031), however; this difference was not seen at one or three months postoperatively. All other ROM and grip strength testing did not show any differences between groups.

**Fig. 2:**
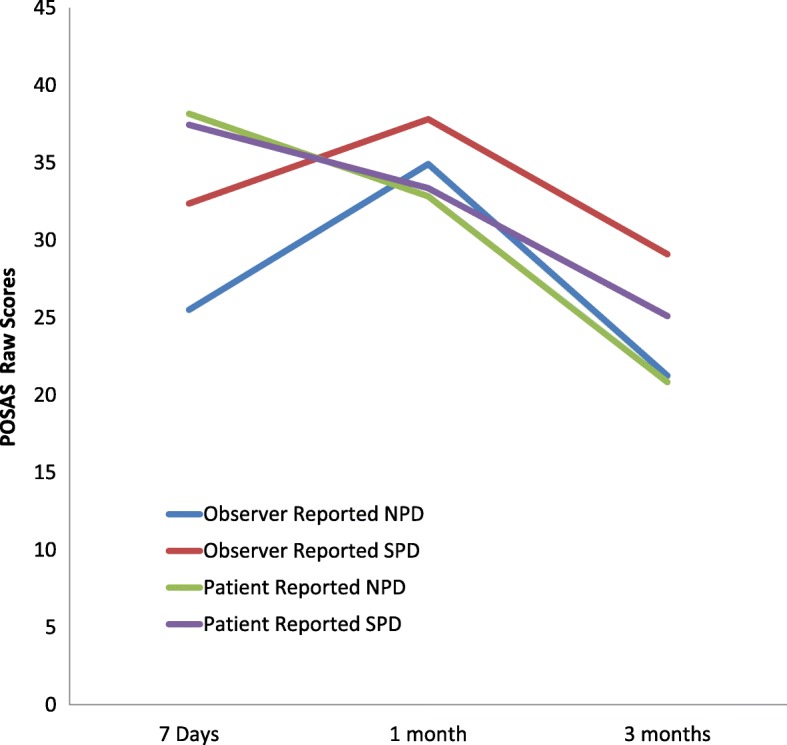
Patient and Observer Scar Assessment Scores (POSAS) following radial forearm free flap reconstruction. SPD – static pressure dressing, NPD – negative pressure dressing

Patients treated with NPD reported better scar aesthetics at seven days, as compared to those in the SPD group (*P* = 0.02). Both patients and observers reported a consistent improvement in scores over three months with lower scores consistent with better scar appearance (Fig. 2).

No differences in graft complications were observed; with 50% of patients in the SPD group experiencing a minor graft complication and 45% of patients in the NPD group (*p* = 0.68). All patients had complete graft take at three months and none required revision in the operating room. There was no effect of smoking, comorbid status, or graft size on wound complications.

## Discussion

With a goal to minimize donor site morbidity, numerous modifications of the RFFF have been developed [13] along with a variety of closure methods including full thickness skin grafts [18], ulnar-based transposition flaps [19], tissue expanders, and autologous tissue. While some of these modifications may offer better cosmesis, some may limit the reconstructive options with shorter pedicles and less flap versatility and others have shown worse functional outcomes at the hand and wrist [20, 21].

Two retrospective studies identified improved rates of STSG healing with NPD as compared to SPD [6, 7]. Vidrine et al. reviewed 45 patients at one and four weeks postoperatively and concluded there were improved rates of graft take in the NPD group with higher rates of major graft loss in the SPD group, though their statistics did not show a significant difference [6]. Andrews et al. found lower than literature rates of graft complications in a retrospective review of patients treated with NPD as compared to SPD [7]. They concluded that NPD should remain in place for six to seven days postoperatively as higher rates of tendon exposure were seen in patients who had their dressings removed sooner.

Additionally, a well designed study by Chio and Agrawal was performed prospectively and compared NPD and SPD [1]. They found no differences in rates of donor site wound complications. They did not perform a formal economic analysis with their results; however, had concerns primarily relating to the additional costs of the dressing as a consideration for the use of NPD as standard practice.

Our study is unique in that it utilizes a smaller, portable, single-use negative pressure device which facilitates early mobilization of the patients’ arm and their overall mobility with no large restrictive vacuum devices. Additionally, we have identified a patient-centered, clinically important difference in hand function during the first postoperative week. The NPD allows patients to be more actively involved in their self-care and facilitates improved communication with retained hand and wrist function. Considering as well, the impact of additional shoulder immobility secondary to spinal accessory nerve injury or neuropraxia as a sequela of neck dissection; the impact of retained hand function could be even more profound and important to help patients manage their own care and recover more quickly. The differences in overall hand function are quite striking at one week postoperatively; and it is possible that this may play a role in earlier involvement in self-care and more rapid discharges from hospital. None of these effects persist beyond the one week follow up and are not likely to impact patients as they continue to recover at home. It is possible that certain patients at higher risk of wound healing complications may benefit most from NPD. The relatively small sample size included in our study did not allow for subgroup analyses examining the impact of specific comorbidities on wound healing complications.

A consistent criticism of NPDs is the additional material costs. Non-healing, chronic wounds are distressing for patients and have a significant impact on their ability to return to their activities of daily living. The cost of the PICO™, the NPD system, is $135 CAD; which is a very minor portion of a one to two week hospital stay as is standard for a head and neck free flap reconstruction. While not part of this study, a formal cost analysis examining the comparative costs of NPD and the potential cost savings relating to wound complications should be undertaken to examine the economic impact for the health care system.

Limitations of the study include the relatively small sample size, which may limit the ability to find statistical significance in some of the secondary analyses. The sex distribution within the groups is unlikely to have affected results; however, there is some evidence that females experience on average more postoperative pain and disability than men, though the differences appear to be small [22].

## Conclusions

Patients treated with NPD for coverage of their STSG following RFFF reconstruction self-report improve hand and wrist function in the immediate post-operative period. Improved hand and wrist mobility following major head and neck reconstruction could facilitate earlier implementation of self-care, more effective communication and improved hand and wrist range of motion following surgery. The additional costs with this single-use, small, portable device are minimal as compared to larger scale vacuum devices and may justify its use with more effective recovery following major head and neck reconstruction.

## Abbreviations

MHQ: Michigan Hand Questionnaire
NPD: Negative pressure dressing
POSAS: Patient and observer scar assessment scale
RFFF: Radial forearm free flap
ROM: Range of motion
SD: Standard deviation
SPD: Static pressure dressing
STSG: Split thickness skin graft
